# Sex-Dependent Rhizosphere Microbial Dynamics and Function in *Idesia polycarpa* through Floral and Fruit Development

**DOI:** 10.3390/microorganisms12102022

**Published:** 2024-10-06

**Authors:** Zhi Li, Qiupeng Yuan, Shasha Wang, Tao Zhang, Yanmei Wang, Qifei Cai, Xiaodong Geng, Yi Yang, Chao Miao, Li Dai, Sohel Rana, Zhen Liu

**Affiliations:** 1 Henan Province Engineering Technology Research Center for Idesia, Zhengzhou 450046, China; lizhi@henau.edu.cn (Z.L.);; 2 National Forestry and Grassland Ad-Ministration Key Laboratory for Central Plains Forest Resources Cultivation, Zhengzhou 450046, China; 3 College of Forestry, Henan Agricultural University, Zhengzhou 450046, China; 4 Key Laboratory of Vegetation Restoration and Management of Degraded Ecosystems, South China Botanical Garden, Chinese Academy of Sciences, Guangzhou 510650, China

**Keywords:** *Idesia polycarpa*, gender specific, rhizosphere bacteria, flowering stage, fruit maturity stage

## Abstract

Male *Idesia polycarpa*, which display distinct morphological and physiological traits, exhibit greater adaptability to stressful environments than females. However, the connection between this adaptability and rhizosphere processes remains unclear. Here, we investigate the differences in root bacterial community structures between male and female plants at different developmental stages, identifying bacterial strains associated with plant sex through functional predictions. This study aims to inform the optimal allocation of male and female plants during cultivation and provide a theoretical basis for sex identification and breeding. Samples from seven-year-old male and female plants were collected during the flowering (May) and fruit ripening (October) stages. Rhizosphere nutrient content and bacterial diversity were analyzed using Illumina high-throughput sequencing technology. The results demonstrate that total nitrogen (TN), total carbon (TC), and available potassium (AK) varied between sexes at different times. No significant differences between male and female plants were observed in the Shannon, Simpson, and Chao1 indexes during the flowering period. However, the Chao1 and Shannon indexes were significantly higher at fruit maturity in male rather than female plants. The predominant phyla of rhizosphere bacteria were *Pseudomonadota*, *Acidobacteriota*, and Actinomycetes. Interestingly, from flowering to fruit ripening, the dominant phyla in both male and female plants shifted from Actinomycetes to *Pseudomonadota*. A significant correlation was observed between pH and AK and rhizosphere bacteria (*p* < 0.05), with metabolism being the main functional difference. This study provides preliminary insights into the functional predictions and analyses of bacteria associated with *Idesia polycarpa*. The above findings lay the groundwork for further investigation into the sex-specific differences in microbial flora across different developmental stages, elucidating the mechanisms underlying flora changes and offering theoretical support for the high-quality management of *Idesia polycarpa*.

## 1. Introduction

About 15,000 species of dioecious plants constitute about 6% of all angiosperms. They are important in ecosystem community structure, functional stability, and biodiversity [1,2,3]. Various studies have shown that dioecious plants of different sexes significantly differ in nutrition, reproduction, and physiology. The difference between male and female plants leads to the phenomenon of proportionality bias of dioecious plants. Most male dioecious plants tend to adapt more to the environment than female dioecious plants [4]. However, the mechanism of their different environmental adaptability has not been explored.

Rhizosphere microorganisms are the most closely related parts of the soil to plants [5]. The rhizosphere microbial community is formed through root–soil interaction. In addition to providing carbon and nitrogen sources for the rhizosphere soil microbial system, the root system can also mediate a plant’s absorption and utilization of soil nutrients and their adaptation to changes in the external environment [6]. Rhizosphere microorganisms can directly affect the availability of soil nutrients to promote plant growth. Soil nutrients can directly regulate the microenvironment of plant roots by changing pH and carbon content, thus affecting the composition and structure of root microbial communities [7].

Plant roots are important for plants to absorb soil nutrients and are the main part of the exchange of nutrients between plants and the soil environment [8]. The interaction between plant roots and rhizosphere microorganisms is highly complex, including positive, negative, and intermediate interactions [9]. Hiltner first described the rhizosphere effect in 1904. When microorganisms are attracted by nutrients secreted from plant roots, the number and activity of microorganisms near plant roots increase. In addition to providing sufficient nutrients, plant roots produce signals that specific microorganisms can recognize to attract colonization [10]. However, the difference in rhizosphere microbial communities between male and female plants is largely unclear. Therefore, exploring the rhizosphere microbial community of dioecious plants may reveal differences between male and female plants in specific environments.

*Idesia polycarpa* is a deciduous species of Idesia in Salicaceae, an excellent afforestation tree species and an excellent woody oil crop, a typical dioecious woody plant [11]. The seeds and fruits of *Idesia polycarpa* can be used for oil extraction. The oil contains many natural active ingredients and can be widely used in medicine, industrial oil, cosmetics, and other fields [12]. The *Idesia polycarpa* is a typical dioecious tree species, and previous studies have found that male plants are more adaptable to stress environments than female plants in terms of morphological and physiological traits [13,14,15]. However, whether this characteristic is related to subsurface rhizosphere processes is unclear. Therefore, in this study, *Idesia polycarpa* was used as experimental material to explore the differences in rhizosphere nutrients, bacterial community structure, and function between male and female plants of *Idesia polycarpa* at the flowering and fruit ripening stages to determine the biological characteristics of male and female plants of *Idesia polycarpa* and to identify the gender-related strains of *Idesia polycarpa*, in order to provide the theoretical basis for field management and gender identification of the *Idesia polycarpa* species.

## 2. Results

### 2.1. Variations in Soil Factors over Time

**Figure 1:**
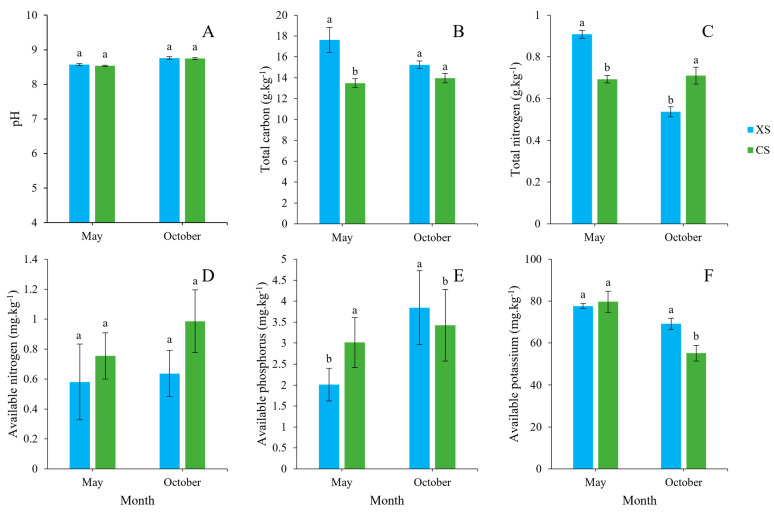
The soil properties of male and female plants of *Idesia polycarpa* at the flowering (May) and fruiting (October) stages. (**A**) Soil pH, (**B**) Total carbon, (**C**) Total nitrogen, (**D**) Available nitrogen, (**E**) Available phosphorus, and (**F**) Available potassium. The lowercase letters indicate significant differences (*p* < 0.05). Data values represent the mean ± SE. Abbreviations: CS, female plant; XS, male plant.

The soil pH in the vicinity of male (XS) and female (CS) plants exhibited an alkaline trend (Figure 1A), and the value was insignificant at both the flowering and fruiting stages. During the flowering stage, male plants demonstrated a notably higher soil total carbon (TC) content than female plants. However, this discrepancy was not evident during the fruit ripening stage (Figure 1B). Similarly, male plants exhibited significantly elevated soil total nitrogen (TN) levels during flowering, whereas female plants surpassed male plants in TN during fruit ripening (Figure 1C). Notably, the available nitrogen (AN) content in the soil surrounding both male and female plants remained relatively low across various growth stages, with no significant disparity observed (Figure 1D). Furthermore, the soil-available phosphorus (AP) content of *Idesia polycarpa* underwent notable variations, with female plants showing higher levels during flowering and male plants exhibiting higher levels during fruit ripening (Figure 1E). The soil available potassium (AK) content did not significantly differ between male and female plants during the flowering stage; however, male plants exhibited significantly higher levels than female plants during fruit ripening (Figure 1F).

### 2.2. OTU Distribution of Rhizosphere Bacteria in Different Periods

**Figure 2:**
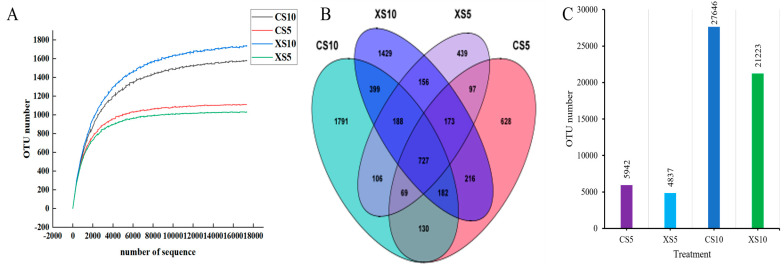
(**A**) Dilution curves of rhizosphere soil samples of *Idesia polycarpa* in different periods, (**B**) rhizosphere bacteria Venn diagram, (**C**) the number of OTUs of rhizosphere bacteria in different periods. Abbreviations: CS5, soil bacteria at the female flowering stage; CS10, soil bacteria at the female fruit ripening stage; XS5, soil bacteria at the male flowering stage; XS10, soil bacteria at the male fruit ripening stage.

The QIIME2 platform was used to perform high-throughput sequencing on the collected samples. The sparse curve of OTU bacterial community richness in different soil samples is shown in Figure 2A. With the increase in sample size, OTU richness tended to be stable. The results indicate that the sequencing depth meets the requirements and can reflect the actual situation of the sample.

The results of the Venn diagram (Figure 2B) showed that 2223 bacterial OTUs were detected in the rhizosphere soil of females, and 1958 in male plants of *Idesia polycarpa* at the flowering stage, and there were 1068 common OTUs between male and female plants. At the fruit ripening stage, 3600 bacterial OTUs were detected in the rhizosphere soil of females, and 3470 in male plants, and male and female plants shared the 1497 common OTUs. The number of OTUs in the soil bacterial community was 727, accounting for about 7%. The number of OTUs unique to CS5, CS10, XS5, and XS10 was 628, 1798, 441, and 1428. Each accounted for 11%, 7%, 9%, and 7% of the total OTUs. In terms of quantity, a certain proportion of bacterial species are common to soils at different times, but there are also unique bacterial communities. Of the four sample groups, the number of bacterial OTUs unique to CS10 was the highest, and the number of bacterial OTUs unique to XS5 was the lowest. In addition, the number of soil bacterial OTUs was distributed in the order CS10 (27,646), XS10 (21,223), CS5 (5942), and XS5 (4837) (Figure 2C). The above results show that the number of OTUs in the rhizosphere soil bacteria at the flowering stage was lower than that at the fruit ripening stage, and the number of OTUs in female plants at different stages was higher than in male plants.

### 2.3. Diversity of Bacterial Communities in Soil Environment at Different Stages

The Alpha diversity index of rhizosphere soil bacteria of male and female plants of *Idesia polycarpa* at the flowering and fruit ripening stages was statistically analyzed. The Chao1 index was used to reflect species richness, and the Shannon and Simpson indexes reflect the diversity of microbial communities. The results show (Table 1) that the α diversity index (except the Simpson index) of bacteria in the rhizosphere soil of *Idesia polycarpa* varied significantly in different months, the richness and diversity of bacterial communities in fruit ripening stage soil samples were higher than those in flowering stage soil samples. The α diversity index of soil bacteria at the flowering stage was CS5 > XS5, and there was no significant difference in the Shannon and Simpson indexes between the two samples. The α diversity index of bacteria in soil at the fruit ripening stage was XS10 > CS10, the Chao1 and Shannon indexes of male plants were significantly higher than those of female plants, and the difference between Simpson index samples was insignificant.

### 2.4. Comparison of Bacterial Community Structure in Soil Environment at Different Times

**Figure 3:**
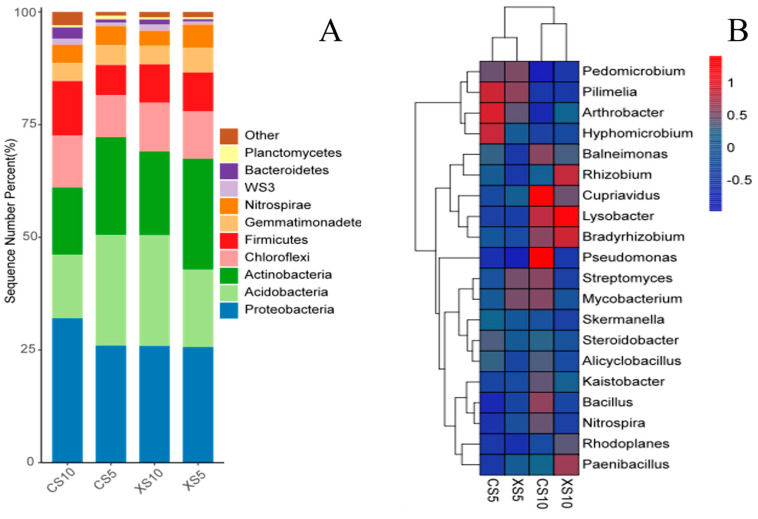
Soil bacteria in different periods in male and female plants of *Idesia polycarpa* represent (**A**) phylum level and (**B**) genus level. Abbreviations: CS5, soil bacteria at female flowering stage; XS5, soil bacteria at male flowering stage; CS10, soil bacteria at female fruit ripening stage; XS10, soil bacteria at male fruit ripening stage.

The bacterial OTU sequences obtained from four groups of soil samples were classified and annotated into 44 phyla and 250 genera. The top 10 phyla with the highest relative abundance were selected to draw a histogram (Figure 3A). At the phylum level, the dominant bacteria in both male and female strains were *Pseudomonadota* (25.64–31.98%), *Acidobacteriota* (14.11–24.56%), and *Actinobateriota* (14.68–24.59%). The relative abundance of the dominant phylum changed in different periods. Compared with the flowering period, the relative abundance of *Acidobacteriota* and *Actinobateriota* in female plants and *Actinobateriota* in male plants decreased significantly in the fruit ripening period. At the fruit ripening stage, the relative abundance of *Acidobacteriota* was significantly different between male and female plants, and it was 1.4 times more abundant in male plants (24.56%) than female plants (14.11%).

The cluster analysis of the top 20 bacterial genera in the relative abundance of the four samples showed that the female and male plants in the same period had similar bacterial community structures and clustered into one group (Figure 3B). In the May soil samples, *Pilimelia, Arthrobacter,* and *Hyphomicrobium* had higher relative abundances in female soil. In the October soil samples, the higher relative abundance genera in female soil included *Pseudomonas* and *Cupriavidus*. The genera with a higher relative abundance in male soil were *Lysobacter* and *Rhizobium*.

### 2.5. Difference Analysis of Rhizosphere Bacteria between Plants of Idesia Polycarpa at Different Periods

**Figure 4:**
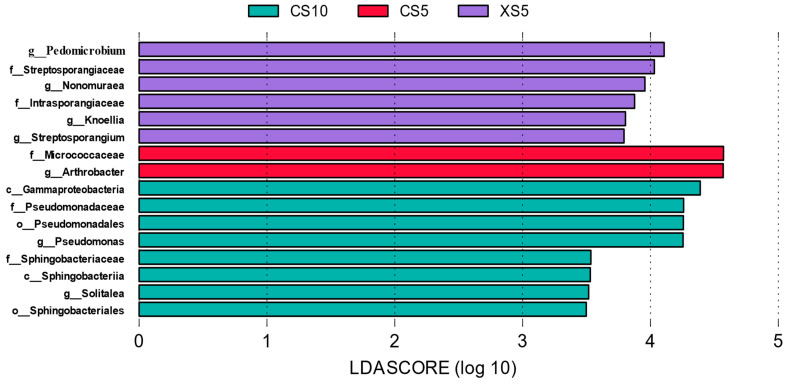
LDA value distribution histogram of bacterial species in the rhizosphere of *Idesia polycarpa* in different periods. Abbreviations: CS5, soil bacteria at the female flowering stage; XS5, soil bacteria at the male flowering stage; CS10, soil bacteria at the female fruit ripening stage; XS10, soil bacteria at the male fruit ripening stage.

In order to further explore species with significant differences between the flowering and fruit ripening stages, LEfSe (LDA effect size) was used to analyze differences in bacterial abundance in the rhizosphere of *Idesia polycarpa* in different growth periods. The LEfSe analysis of rhizosphere bacteria (Figure 4) showed that the XS5 sample had six different indicator species, of which *Pedomicrobium* was the most significant taxonomic unit. There were two differential indicator species in the CS5 samples, of which *Arthrobacter* and *Micrococcaceae* were the most significant taxa. Additionally, there were eight differential indicator species in the XS5 samples, among which *Gamma Pseudomonadota*, *Pseudomonadales,* and *Pseudomonas* were the most significant taxa.

### 2.6. Correlation Analysis between Soil Factors and Bacterial Community Structure

**Figure 5:**
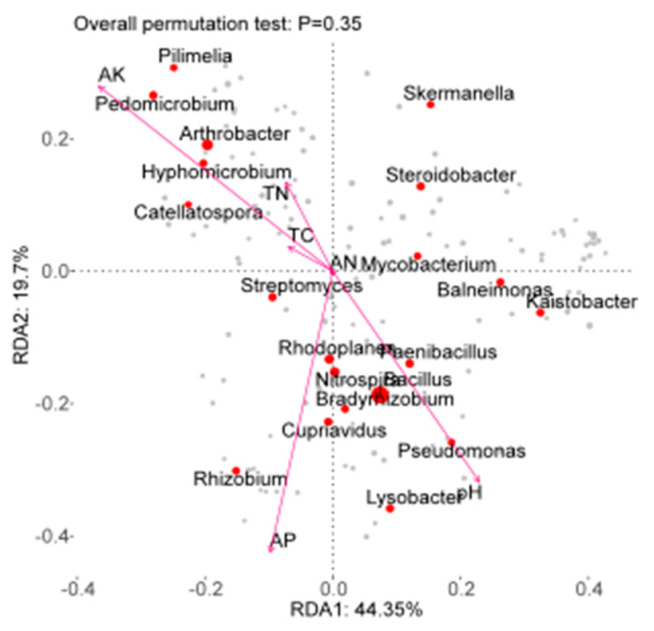
Correlation analysis between soil factors and bacterial community structure. Abbreviations: pH, power of hydrogen; TC, total carbon; TN, Total nitrogen; AP, available phosphorus; AK, available potassium.

In order to analyze the key factors influencing bacterial community changes in the soil environment under different rotation patterns, an RDA analysis was performed on soil fungal community structure and environmental factors such as pH, TC, and TN (Figure 5). The first and second axes accounted for 64% of the variables, and the two axes may reflect the influence of soil environmental factors on soil bacterial communities. The RDA results (Table 2) show that AK (*r* = 0.766, *p* = 0.003), pH (*r* = 0.653, *p* = 0.005), and AP (*r* = 0.724, *p* = 0.003) were the key factors leading to differences in soil bacterial communities.

**Figure 6:**
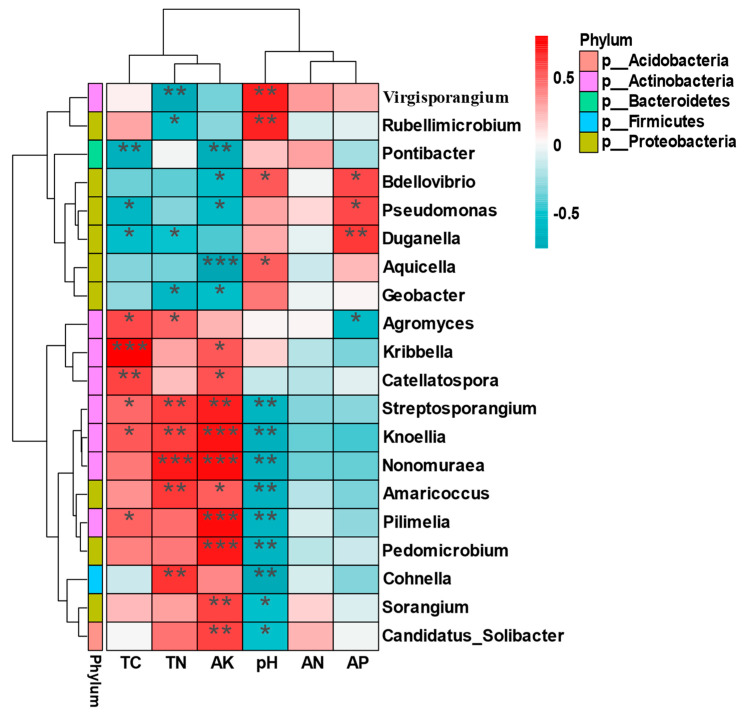
Cluster analysis of soil nutrients and rhizosphere bacteria in different periods of male and female plants, through comparison with the KEGG database. Abbreviations: TC, total carbon; TN, total nitrogen; AK, available potassium; pH, power of hydrogen; AN, available nitrogen; AP, available phosphorus. A single asterisk represents * *p* < 0.05; a double asterisk represents ** *p* < 0.01; and a triple asterisk represents *** *p* < 0.001 significance value.

In order to further explore the relationship between soil bacterial community composition and soil environmental factors, Spearman correlation analysis was performed on the top 20 bacterial genera and soil physical and chemical factors (Figure 6). The results show that AK and pH were significantly correlated with most genera (*p* < 0.05/0.01/0.001). Additionally, the TC and TN were significantly positively correlated with most *Actinobateriota* bacteria and negatively correlated with some bacteria in *Pseudomonadota*. In addition, no correlation was found between AN and soil bacterial community composition.

### 2.7. Functional Differences in Rhizosphere Bacteria between Male and Female Plants of Idesia Polycarpa in Different Periods

PICRUSt2 software was used to predict the metabolic function of soil bacterial communities, and the results show gene sequence annotation of all bacterial flora with a relative abundance > 1% in the rhizosphere of male and female plants of *Idesia polycarpa* in the primary functional metabolic pathway. The functions annotated in the gene sequence of all bacterial flora were divided into six categories. These are metabolism, environmental information processing, genetic information processing, cellular processes, human diseases, and organismal systems. Among them, metabolism is the most important function of soil bacteria, accounting for 74.54%~79.51% of the total functional classification. A total of 47 sub-functions were annotated in the secondary pathway, and sub-functions with a relative abundance of more than 1% of the total abundance were selected for statistical purposes (Table S1). The bacterial community functions of male and female plants in different periods were similar, but the abundance of sub-functional pathways was quite different, including cell growth and death (1.94–2.06%).

Cell movement (2.27–2.63%) and cell community prokaryotes (1.33%~1.42%) in the cell process showed that the abundance of sub-functions at the fruit ripening stage was higher than that at the flowering stage, and higher for male plants than for female plants. Membrane transport (1.01%~1.32%) in environmental information processing was higher in the fruit ripening stage than in the flowering stage and higher in males than females. Folding, classification and degradation (2.83%~3.14%), replication and repair (4.24%~4.61%), and translation (2.51%~2.86%) in genetic information processing were higher in the fruit ripening stage than in the flowering stage, and higher for male plants than for female plants. Antimicrobial resistance (1.44%~1.55%) in human diseases showed that the abundance of sub-functions in the fruit ripening stage was higher than that in the flowering stage and that in male plants it was higher than in female plants. There were 13 metabolic subfunctions, which were relatively abundant in each sample. Among them, the chemical structure conversion map (1.54%~1.88%) showed that metabolic subfunctions were higher in male plants than female plants at the fruit ripening stage, and higher in female plants than male plants at the flowering stage. Amino acid metabolism (11.60%.~12.50%), carbohydrate metabolism (9.58%~10.36%), lipid metabolism (6.03%~6.35%), and other 12 metabolic functions were higher in male plants than in female plants at the fruit ripening and flowering stages.

**Figure 7:**
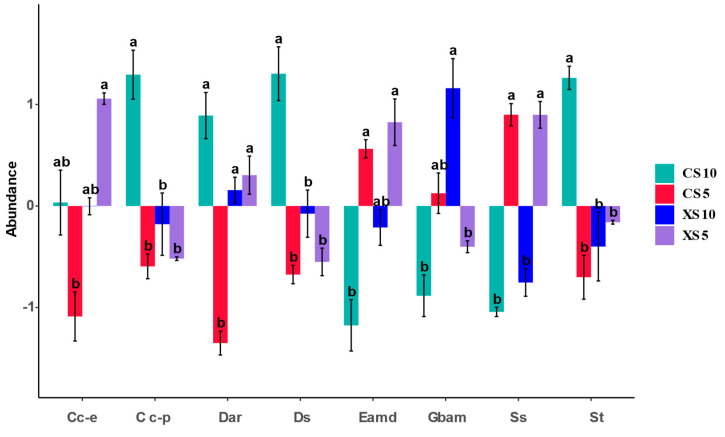
Differences in metabolic sub-functions between male and female plants at different stages (Cc-e: cellular community—eukaryotes, Cc-p: cellular community—prokaryotes, Dar: development and regeneration. Ds: digestive system, Eamd: endocrine and metabolic disease, Gbam: glycan biosynthesis and metabolism, Ss: sensory system, St: signal transduction). The lowercase letters indicate significant differences (*p* < 0.05) among each cellular community’s female and male flowering and fruiting stages. Data values represent the mean ± SE. Abbreviations: CS5, soil bacteria at the female flowering stage; XS5, soil bacteria at the male flowering stage; CS10, soil bacteria at the female fruit ripening stage; XS10, soil bacteria at the male fruit ripening stage.

The abundance of data on existing metabolic pathways was analyzed, and a histogram was drawn using multiple comparisons of the Dunn Test. The results show eight sub-functions with significant differences between male and female plants in different periods (Figure 7). There were significant differences in drug resistance function, such as to antineoplastic drugs, between male and female plants. The function of bacterial prokaryotes (drug resistance: antimicrobial) in the female fruit ripening stage was significantly different from that in the male flowering and fruit ripening stages. The development and repair function of rhizosphere bacteria in the female flowering period significantly differed from that in the female fruit ripening period and male flowering period and fruit ripening stage. The digestive system and signal transduction function of female plants at the fruit ripening stage significantly differed from those of male plants at the flowering and fruit ripening stages. The function of endocrine and metabolic disease in the female fruit ripening stage significantly differed from that in the male and female flowering stages. The function of Glycan biosynthesis and metabolism in mature male plants significantly differed from that of male flowering stage and ripening female plants. The sensory system of male and female plants at the flowering stage was significantly different at the fruit ripening stage.

## 3. Discussion

### 3.1. Structure and Diversity of the Bacterial Community in Idesia Polycarpa Soil in Different Periods

In this study, the data show that the bacterial community structure of male and female plants of *Idesia polycarpa* was significantly different in different periods. The number of OTUs from high to low (CS10 > XS10 > CS5 > XS5) indicates that the fruit ripening period had more OTUs than the flowering period, which was similar to other studies [16]. The number of OTUs in female plants was greater than in male plants at different stages, which is also true of poplar plants [17]. Further species annotation found that the main composition of microbial communities in different periods of *Idesia polycarpa* was different, and the relative abundance of different types of bacteria changed in taxonomy. The bacterial community in the rhizosphere soil of *Idesia polycarpa* is mainly composed of *Pseudomonadota*, *Acidobacteriota*, and *Actinobateriota*. These dominant bacteria are enriched in the rhizosphere environment and are related to their environmental solid adaptability [18]. The change in growth period significantly affected the dominant microorganisms in the rhizosphere soil of male and female plants. In both periods, the dominant bacteria in the rhizosphere soil of male and female plants changed from actinomycetes to *Pseudomonadota*. *Actinobateriota* has complex metabolic systems and can produce a variety of bioactive metabolites, which have significant application value in promoting root growth and improving antibacterial ability and stress resistance [19]. The increase in temperature and rainfall in May is the high incidence period of plant diseases, which may be the main reason for the dominant bacteria in the rhizosphere soil of male and female plants of *Idesia polycarpa* at the flowering stage. In October, when trees store nutrients, *Pseudomonadota* contributes to soil processes by decomposing and transforming organic matter [20], degrading cellulose and lignin [21], and serving as key functional groups for soil carbon and nitrogen fixation [22]. The increase in its relative content in October is conducive to accumulating substances in the male and female trees of *Idesia polycarpa*, laying a foundation for the following year’s growth. In addition, the number of dominant bacterial genera in the rhizosphere soil of female plants was higher than that of male plants, which was related to differences in physiological metabolism between male and female plants at different growth and development stages.

### 3.2. Effects of Soil Environmental Factors on the Composition and Structure of Soil Fungal Communities

Changes in different periods will cause changes in environmental factors such as soil pH, soil carbon, and nitrogen nutrient content [23], and the composition and structure of bacterial communities in the soil environment are usually closely related [24]. In this study, through RDA redundancy analysis, it was found that soil pH, AP, and AK are important nutrient elements that affect the bacterial community in the soil environment [25]. Over half of the top 20 bacterial genera showed significant correlations with soil pH, greatly influencing bacterial community structure and composition. This is consistent with Zhou’s findings [26], which demonstrated that changes in pH can directly affect soil microbial community structure. Our study also found that the bacterial community diversity index significantly correlated with AP and AK. The dominant phylum shifted from Actinobacteriota during the flowering stage to *Pseudomonadota* at the fruit ripening stage. Wang [27] confirmed that soil-available phosphorus and potassium content can significantly alter the diversity of the *Pseudomonadota* bacterial community. As the developmental stages of *Idesia polycarpa* progress, changes in soil phosphorus and potassium levels lead to shifts in the dominant phyla of rhizosphere bacteria in *Idesia polycarpa*.

### 3.3. Functional Differences in Rhizosphere Bacteria between Male and Female Idesia Polycarpa in Different Periods

The analysis of the relative abundance of functional bacterial genes in rhizosphere soil samples from different developmental stages of *Idesia polycarpa* suggests that metabolic pathways consistently had the highest relative abundance, highlighting metabolism as a key factor in the plant’s growth and development. Eight sub-functions showed significant differences between male and female plants at various stages. Notably, glycan synthesis and metabolism influence carbohydrate production [28]; endocrine and metabolic pathways are closely linked to plant disease resistance and stress tolerance [29]; digestive and sensory systems affect the nutritional balance in plants [30]; and signal transduction plays a role in communication, material circulation, and energy exchange between the plant and its environment [31]. As the PICRUSt functional prediction analysis provides only a preliminary insight into the functions of associated bacteria, further research is needed to verify the differential bacterial communities in male and female plants during different stages of *Idesia polycarpa* development.

## 4. Materials and Methods

### 4.1. Test Site

The experimental site is located at the Forestry Experimental Station of Henan Agricultural University in Zhengzhou, Henan Province (112° 42′ 114° 14′ E and 34° 16′ 34° 58′ N), China. The area has a temperate continental monsoon climate and is the boundary between the middle and lower reaches of the Yellow River. The altitude of the experimental site is 97 m, the annual average temperature is 14.3 °C, the annual precipitation is 623.30 mm, mainly concentrated from June to September, and the frost-free period of the year is 220 days. Soils in Zhengzhou are characterized by low organic matter content, high effective phosphorus content, and coarse texture [32]. The soil texture of this trial site is sandy loam.

### 4.2. Experimental Materials and Design

The experimental materials consisted of seven-year-old *Idesia polycarpa* trees cultivated at Henan Agricultural University, with a plant spacing of 1.5 × 1.5 m. Male and female (with three replicates) *Idesia polycarpa* trees exhibiting healthy and similar growth were selected for the study. Rhizosphere soil samples were collected using the root drill method. The soil was obtained from four directions (southeast, southwest, northeast, and northwest) around each tree at a depth of 20–40 cm, specifically focusing on the soil within 4 mm of the root surface [33]. The four samples from each tree were thoroughly mixed to form a composite rhizosphere soil sample. The collected soil samples were then sealed in sterilized sampling bags and stored in a cooler for preservation until further analysis [34].

The soil pH was determined by weighing 10 g of soil (accurate to 0.01 g) and placing it in 50 mL of boiling water. Then, 25 mL of CO_2_-free water was added to the mixture, maintaining a soil-to-liquid ratio of 1:2.5. The solution was stirred with a glass rod for 1 min to disperse the soil particles fully. After 30 min, the pH was measured using an acidimeter (LC-PH-3S, Shanghai LiChen Bangxi Instrument Equipment Co., Shanghai, China). The pH was recorded from the upper layer of the sample, with each measurement repeated three times to obtain an average value.

Soil alkaline nitrogen (AN) was determined using the alkaline diffusion method, where soil samples were treated with 1.8 M NaOH and Zn-FeSO_4_ reductant and titrated with a 0.1 M HCl standard solution. Total carbon (TC) and total nitrogen (TN) were measured using an automated elemental analyzer (Euro Vector EA3000, Shanghai Wolong Instrument Co., Ltd., Shanghai, China). Available phosphorus (AP) was quantified by a 0.5 M NaHCO_3_ leaching method, followed by molybdenum antimony resistance colorimetry. In contrast, available potassium (AK) was measured using the same leaching method and a flame photometer. Total phosphorus (TP) and total potassium (TK) were analyzed through the H_2_SO_4_-H_2_O_2_ digestion method, with TP determined by molybdenum antimony resistance colorimetry and TK by flame photometry.

### 4.3. DNA Extraction and PCR Amplification

The total DNA of rhizosphere bacteria was extracted and purified using the E.Z.N.A.^®^ soil kit (Omega Bio-Tek, Norcross, GA, USA), and the purity and concentration of the extracted DNA were measured using a NanoDrop 2000 spectrophotometer. The quality of the extracted DNA was then verified through 1% agarose gel electrophoresis [35]. PCR products were recovered from a 1% agarose gel, and the V3-V4 region of the bacterial 16S rRNA gene was amplified using the primers 338F (5′-ACTCCTACGGGAGGCAGCAG-3′) and 806R (5′-GGACTACHVGGGTWTCTAAT-3′). Following a series of purification steps, including elution, electrophoresis detection, and quantitative analysis, the purified PCR fragments were used to construct PE2 × 300 libraries according to the operating standards of the Illumina MiSeq platform (Illumina, San Diego, CA, USA) [36]. Once the library was constructed and validated, sequencing was performed on the Illumina MiSeq PE300 platform. The DNA extraction, PCR amplification, and sequencing for this study were performed by a specialized company, Wekemo Tech Group Co., Ltd. (Shenzhen, China), ensuring precision and expertise.

### 4.4. Sequencing Information Statistics and Data Analysis

The original FASTA sequence file was modified and imported into a format compatible with QIIME2 using the import plugin tools. The QIIME2 dada2 plugin was then employed for quality control, trimming, denoising, splicing, and chimera removal. This process generated the final feature sequence table. The QIIME2 feature-classifier plugin was employed to categorize the representative ASV sequences using the GreenGenes 13_8 database, trained at 99% similarity, resulting in a detailed species classification table. The QIIME2 feature-table plugin was also used to remove contaminating mitochondrial and chloroplast sequences [37]. The QIIME2 core-diversity plugin calculated the diversity matrix and Alpha diversity metrics, including the Chao1 index, Shannon index, and the number of observed OTUs to analyze diversity further. Beta diversity was assessed using Bray–Curtis, unweighted UniFrac, and weighted UniFrac indexes to compare microbial community structure differences across samples, with PCA plots illustrating sample diversity. Statistical analyses of the Alpha diversity index and rhizosphere bacterial species, including significance and correlation testing, were conducted using IBM SPSS v. 26 (IBM Corp., Armonk, NY, USA, https://www.ibm.com/, accessed on 30 November 2023) to evaluate differences between treatment groups.

## 5. Conclusions

The study revealed distinct differences in soil nutrient content, rhizosphere bacterial composition, diversity, and function between male and female *Idesia polycarpa* plants during the flowering and fruit ripening stages. The AP levels showed significant variation between male and female plants at the flowering stage. In contrast, TN, AN, and AK differed significantly during the fruit ripening stage. The dominant bacterial phyla also shifted, with *Actinobateriota* prevailing at the flowering stage and *Pseudomonadota* at the fruit ripening stage. Additionally, female plants exhibited a significantly higher number of dominant bacterial species in their rhizosphere than male plants. Functional predictions further indicated that the ecological roles of soil bacteria differed between male and female plants across developmental stages, with notable differences in metabolic functions between the flowering and fruiting periods. These findings provide valuable insights into the adaptive mechanisms of dioecious plants during reproductive stages and offer a scientific basis for improving the field management of *Idesia polycarpa*.

## Figures and Tables

**Table 1:** α diversity index of bacterial community in rhizosphere soil of male and female plants of *Idesia polycarpa*. Abbreviations: CS5, soil bacteria in female flowering stage; XS5, soil bacteria at male flowering stage; CS10, soil bacteria in female fruit ripening stage; XS10, soil bacteria at male fruit ripening stage.

Period	Treatments	Chao1 Index	Shannon Index	Simpson Index
May bacteria	CS5	1129.49 ± 48.28 ^a^	9.57 ± 0.05 ^a^	0.998 ± 0.0001 ^a^
	XS5	1050.96 ± 16.78 ^ab^	9.46 ± 0.05 ^a^	0.9978 ± 0.0003 ^a^
October bacteria	CS10	1636.21 ± 29.22 ^b^	9.84 ± 0.12 ^b^	0.9975 ± 0.0006 ^a^
	XS10	1808.26 ± 7.9 ^a^	10.08 ± 0.07 ^a^	0.9982 ± 0.0002 ^a^

Note: The lowercase letters indicate significant differences (*p* < 0.05) in each index’s female and male flowering and fruiting stages.

**Table 2:** Data table of soil nutrients in RDA analysis.

Parameter	r^2^	*p*-Value
TC	0.131	0.535
TN	0.255	0.293
AN	0.008	0.951
AP	0.724	0.103
AK	0.766	0.001
pH	0.653	0.005

## Data Availability

Data will be made available on request.

## Supplementary Materials

The following supporting information can be downloaded at: https://www.mdpi.com/article/10.3390/microorganisms12102022/s1. Table S1. KEGG database main metabolic pathway abundance.
