# Supplementary material for: Sex-Dependent Rhizosphere Microbial Dynamics and Function in Idesia polycarpa through Floral and Fruit Development
Source: Microorganisms. 2024 Oct 6;12(10):2022. doi: 10.3390/microorganisms12102022 (PMC11509645; doi:10.3390/microorganisms12102022)
Supplement: Supplementary file 1 [file microorganisms-12-02022-s001.zip › microorganisms-3236593-supplementary.pdf]

Table S1. KEGG database main metabolic pathway abundance.

Metabolism pathway		Abundance			
L1	L2	CS10	CS5	XS10	XS5
Cellular Processes	Cell growth and death	26873.69	12150.26	28910.67	12039.24
	Cell motility	36310.59	14244.54	34121.86	14931.62
	Cellular community – prokaryotes	19497.74	8332.51	20025.70	8289.48
Environmental Information Processing	Membrane transport	17976.17	7766.45	18691.74	7738.37
Genetic Information Processing	Folding, sorting and degradation	38992.52	18440.75	43145.00	18375.51
	Replication and repair	57772. 40	26803.12	63467.82	26932.12
	Translation	34629.49	16825.87	39275.22	16703.10
Human Diseases	Drug resistance: antimicrobial	20121.76	9038.08	21459.91	9083.38
Metabolism	Amino acid metabolism	159688.96	74001.98	173979.81	72943.63
	Biosynthesis of other secondary metabolites	81448.98	38441.02	91019.60	37886.10
	Carbohydrate metabolism	131907.25	61232.12	144962.23	60499.24
	Chemical structure transformation maps	22674.67	10078.67	22943	11011
	Energy metabolism	57770.34	26038.99	61945.78	25963.35
	Global and overview maps	81160.27	37623.51	89010.41	37264.21
	Glycan biosynthesis and metabolism	37631.04	17578.92	42713.79	17215.50
	Lipid metabolism	86308.17	37768.97	91236.89	37107.33
	Metabolism of cofactors and vitamins	134863.89	62578.18	147182.04	62351.25
	Metabolism of other amino acids	92772.45	42799.38	100947.37	42104.32
	Metabolism of terpenoids and polyketides	50179.38	22691.88	53382.85	22642.19
	Nucleotide metabolism	17299.15	8100.24	19104.00	8056.28
	Xenobiotics biodegradation and metabolism	71993.15	30064.28	72714.99	28950.01
